# 

**Published:** 1840-07

**Authors:** 


					. ?, m
THE
BRITISH AND FO
MEDICAL RE
OB
QUARTERLY JOURNAL
PRACTICAL MEDICINE AND SURGERY
LIBRARY OF THE
Orleans Parish Medical feisty
EDITED BY
JOHN FORBES M.D. F.R.S.
VOL. X.
JULY?OCTOBER 1840
LONDON
JOHN CHURCHILL PRINCES STREET SOHO.
London: Printed by C. Adlarp,
Bartholomew Close.

				

